# Epistatic interactions of genetic loci associated with age-related macular degeneration

**DOI:** 10.1038/s41598-021-92351-4

**Published:** 2021-06-23

**Authors:** Christina Kiel, Christoph A. Nebauer, Tobias Strunz, Simon Stelzl, Bernhard H. F. Weber

**Affiliations:** 1 Institute of Human Genetics, University of Regensburg, Franz-Josef-Strauss-Allee 11, 93053 Regensburg, Germany; 2 Institute of Clinical Human Genetics, University Hospital Regensburg, Regensburg, Germany

**Keywords:** Functional genomics, Gene expression, Gene regulation, Genetic association study, Genetic interaction

## Abstract

The currently largest genome-wide association study (GWAS) for age-related macular degeneration (AMD) defines disease association with genome-wide significance for 52 independent common and rare genetic variants across 34 chromosomal loci. Overall, these loci contain over 7200 variants and are enriched for genes with functions indicating several shared cellular processes. Still, the precise mechanisms leading to AMD pathology are largely unknown. Here, we exploit the phenomenon of epistatic interaction to identify seemingly independent AMD-associated variants that reveal joint effects on gene expression. We focus on genetic variants associated with lipid metabolism, organization of extracellular structures, and innate immunity, specifically the complement cascade. Multiple combinations of independent variants were used to generate genetic risk scores allowing gene expression in liver to be compared between low and high-risk AMD. We identified genetic variant combinations correlating significantly with expression of 26 genes, of which 19 have not been associated with AMD before. This study defines novel targets and allows prioritizing further functional work into AMD pathobiology.

## Introduction

A first successful genome-wide association study (GWAS) was reported in 2005 and identified with genome-wide significance genetic variants at the CFH locus associated with age-related macular degeneration (AMD), a complex disease which is a frequent cause of progressive vision loss in the elderly population^1^. Since then, the list of AMD-associated genetic variation has grown exponentially, presently bringing the total to 52 independent common and rare variants across 34 chromosomal loci^2^. In an initial effort to extract biological meaning from the latter association data, enrichment analyses were done to determine molecular pathways from the 368 genes located within the immediate AMD loci as defined by index variants and proxies at r^2^ ≥ 0.5 within ± 100 kb of the lead signal. This broadly emphasized the lipid metabolism, extracellular matrix organization and assembly as well as the complement pathway to play a critical role in AMD pathogenesis^2^.

A more refined dissection of the biological mechanisms triggering AMD disease is challenging, although it is a crucial step to translate genetic findings into a clinical context. Understanding the functional impact of synonymous or non-synonymous variants in exonic sequences appears rather straightforward as they likely exert a direct effect on protein translation or stability^3^. However, in AMD over 90% of associated genetic variation is estimated to be located in intronic or intergenic regions^2^, which hampers an intuitive access to understanding their pathological impact. A growing number of studies now suggests that variation in noncoding regions likely affect mRNA expression of nearby or distal genes through promoter, enhancer or silencer effects, or modulate transcription factor binding affinities, alternative splicing, and/or other epigenetic modifications^4^. To some extent, such an altered gene expression can be addressed by mapping expression quantitative trait loci (eQTL)^5^. In AMD, eQTL analyses were conducted in several tissues. For example, Ratnapriya and colleagues investigated eQTL in the retina, and detected altered gene expression for 20 potentially AMD-associated genes^6^, while in liver a total of 15 genes were identified^7^. Further, a transcriptome-wide association study included 27 different tissues and identified 106 genes with an expression profile linked to the genetic risk of AMD^8^. Although primary pathology of AMD manifests in the back of the eye, eQTL findings in various tissues reveal genes potentially influencing general tissue integrity and homeostasis^9–11^. This is strengthened by pleiotropy analyses, demonstrating a common genetic basis of AMD and several other phenotypes, such as cardiovascular diseases or metabolic traits^12,13^. Although the retinal pigment epithelium (RPE) may be the cell type of interest in AMD pathology^14^, it is likely that circulating molecules ultimately contribute to local manifestations^15–17^. Against this background, liver tissue is of particular interest as it is the major expression site for secreted proteins involved in at least two major pathways of AMD pathology, including the lipid metabolism and the complement system^18–20^. Finally, a gene expression dataset with nearly 600 individuals is available for liver tissue providing sufficient statistical power to perform advanced analyses.

So far, eQTL mapping in AMD was directed exclusively on studying effects of single genetic variants^6,7^. Additive models including the integration of combinations of genetic variation on gene expression have not been considered. Here, we focused on genetic variation in loci which were assigned to one of three likely biological pathways in AMD aetiology, including lipid metabolism, extracellular matrix organization, and the complement cascade^2,9^. We combined customized genetic risk scores (GRS) and eQTL mapping and evaluated an influence on gene expression in liver tissue for genetic variants individually or in combination with each other. Such an approach more closely resembles a true biological situation, where AMD patients likely harbor multiple risk variants with possible synergistic or antagonistic effects on a defined outcome. Taken together, exploiting possible epistatic interactions of AMD-associated variants on gene expression regulation has revealed at least 19 genes of interest which have not been found in the available AMD datasets so far. This could open new avenues to learn about biological pathways and mechanisms underlying AMD disease.

## Results

### Defining pathway-related clusters of independent AMD-associated variants

**Figure 1:**
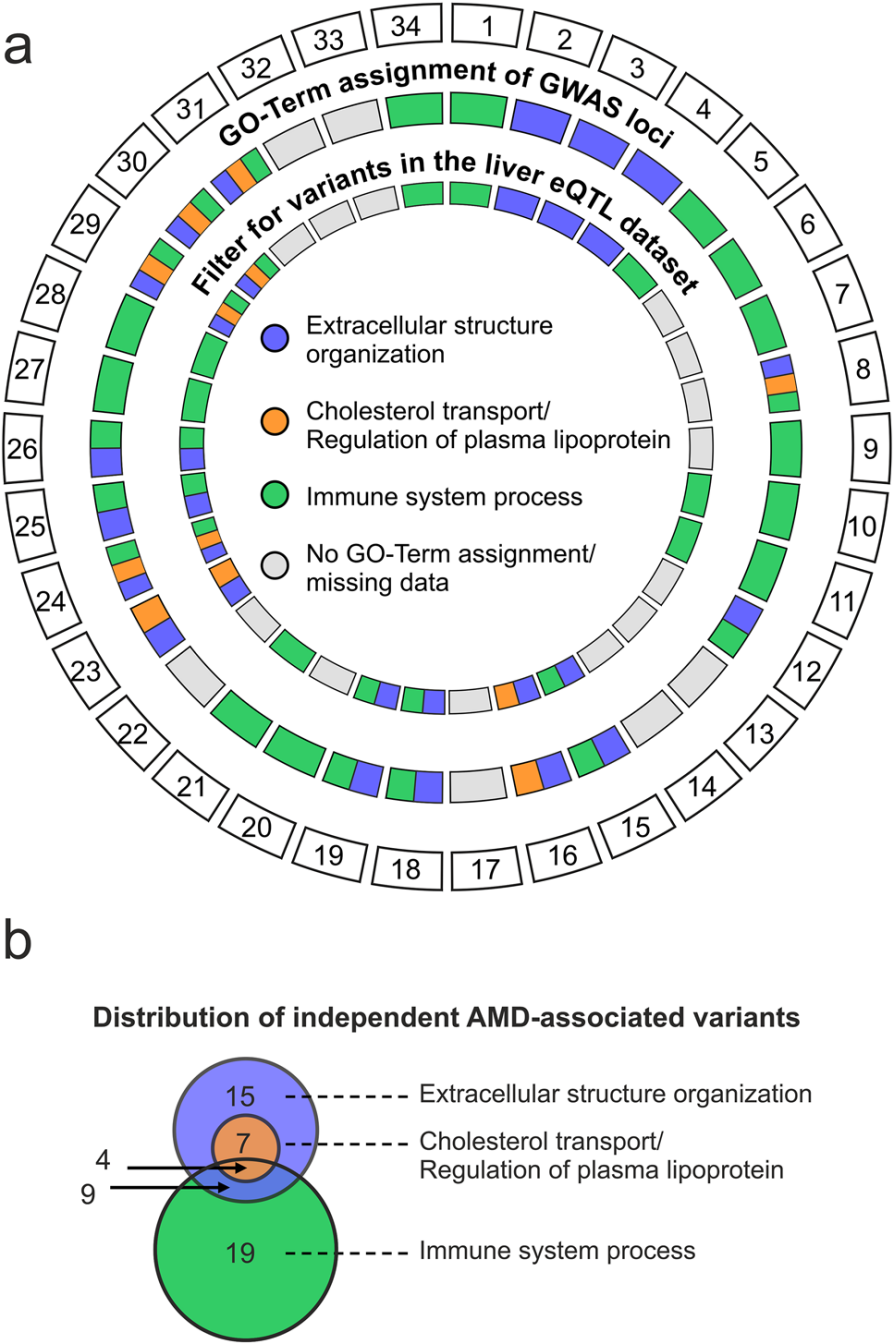
Categorizing independent AMD-associated variants. (**a**) AMD-associated loci as defined in ref. 2 were assigned to three biological pathways discussed to be relevant in AMD pathobiology, particularly the “Extracellular structure organization”, “Cholesterol transport” and “Regulation of plasma lipoprotein”, and “Immune system process”. A locus was assigned to a pathway if a gene located in this locus (locus definition: index variant(s) and proxies (r^2^ > 0.5; +/- 100 kb)^2^ is functionally associated by GO-Terms. Subsequently, the independent signals within these loci were correlated with the liver eQTL dataset reported by Strunz et al.^7^. (**b**) Distribution of independent AMD-associated variants over the three biological pathways identified. Overlapping circles indicate independent variants that were assigned to more than one category. Details are given in Supplementary Table S1.

To search for epistatic effects of independent AMD-associated genetic variants on gene expression in liver tissue, we first selected tentative biological routes to disease mechanisms represented by three likely AMD-associated pathways including the lipid metabolism, extracellular matrix organization, and the complement cascade^2^. We used the g:Profiler database^21^ to assign gene ontology terms (GO-terms) to the 386 genes within the 34 genome-wide associated AMD loci as defined by Fritsche et al. (index variants and proxies at r^2^ ≥ 0.5 within ± 100 kb of lead variants)^2^. GO-terms used for locus categorization were “cholesterol transport” (GO:0030301), “regulation of plasma lipoprotein” (GO:0097006), “extracellular structure organization” (GO:0043062), and “immune system process” (GO:0002376). Loci were assigned to multiple pathways, if the corresponding genes mapped to several GO-terms. In total, seven of the 34 independent AMD loci were assigned to the lipid metabolism pathway, 16 to the extracellular matrix term, and 23 to the complement system (Fig. 1a). Six of the 34 AMD loci were not assigned to any of the selected GO-terms.

We then filtered the liver eQTL dataset reported by Strunz et al.^7^ for independent lead variants within the 28 pathway-associated AMD loci. This resulted in 21 loci for further analysis, corresponding to 31 independent genetic variants. Specifically, seven variants at 5 loci were assigned to the lipid metabolism pathway, 15 variants at 13 loci to the extracellular matrix pathway, and 19 variants at 16 loci to the complement system pathway (Fig. 1b and Supplementary Table S1). Interestingly, the five loci assigned to the lipid metabolism pathway are also associated with the extracellular matrix pathway.

### Defining test parameters in liver tissue

**Figure 2:**
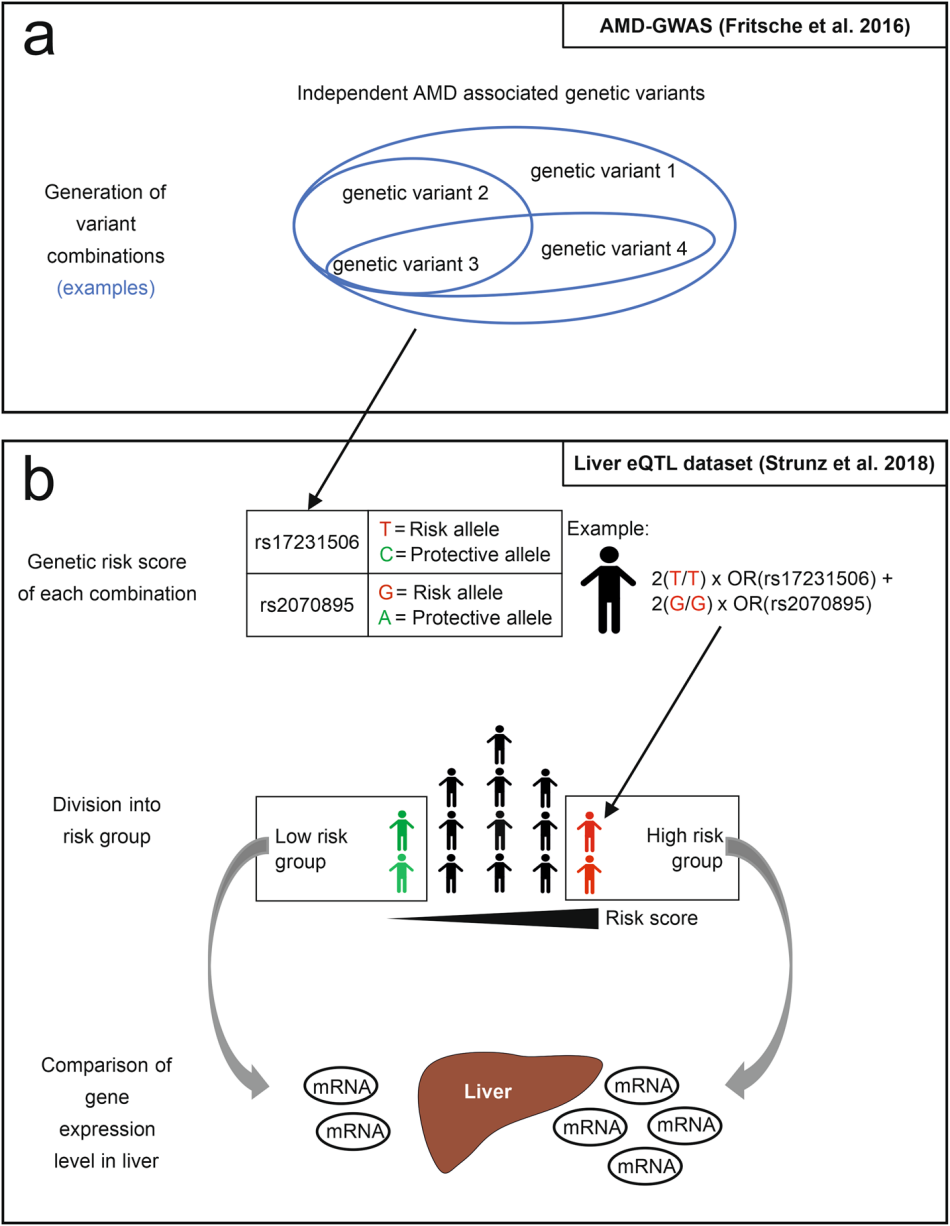
Schematic project workflow. (**a**) AMD-associated genetic variants were used to generate unique variant combinations. (**b**) Based on each combination, a genetic risk score (GRS) was calculated for 588 individuals represented in the liver eQTL dataset^7^. According to the individual genetic risk profile, each person was graded into risk groups, with a low-risk group containing the 30%-quantile of the lowest scores and a high-risk group containing the 30%-quantile of the highest scores. Consequently, expression of 24,123 genes in liver tissue were compared between the risk groups.

The category “lipid metabolism” was used to initially define the test parameters for the identification of significant variant combinations (eCombinations) with a joint effect on gene expression regulation in liver tissue. In total, 5 loci containing 7 independent variants were included in this category (Fig. 2a). The 7 variants were organized in 127 unique combinations.

Effects on the regulation of gene expression were determined based on the GRS approach, which categorizes individuals into low and high-risk groups for the respective variant combination. Subsequently, gene expression was compared between low and high-risk individuals (Fig. 2b). Group size was defined based on the ability to detect differently expressed genes, which were previously reported to be influences by AMD-associated variants (eGenes)^7^. For this purpose, a GRS including all seven variants assigned to the “lipid metabolism” category was calculated. Afterwards, different risk group sizes, ranging between 5 and 35% of the corresponding lowest or highest risk quantiles, were tested for the ability to detect four known eGenes, including *LIPC*, *CETP*, *ALDH1A2* and *ADAM10*^7^, after correction for multiple testing by a false discovery rate (FDR, Q-value) smaller than 5%. Consequently, low and high-risk groups were defined at the 30% highest and lowest quantiles, respectively (Supplementary Figure S1). These two groups each contain 176 liver tissue samples.

To define the significance threshold, we considered the expression of 24,123 genes for 127 combinations requiring over 3 million tests in the category “lipid metabolism” (Table 1). Quantile–quantile plots with all P-values of tests performed in the respective categories allow adjustment for multiple testing (Supplementary Figure S2a). We defined the FDR to be under 5%, as this threshold produces comparable results to the thresholds that can be derived from the quantile–quantile plots.

**Table 1:** Statistical overview of variant interactions investigated.

	Lipid metabolism pathway	Extracellular matrix pathway	Immune pathway
Independent signals^a^	7	15	19
Combinations	127	32,767	524,287
Tests (24,123 genes)	3 M	790 M	12.6 B
Significance threshold	Q-value < 0.05	Q-value < 0.05	P-value < 10^–5^
Significant results	2	0	641,016
eUnique eCombinations	2	0	326,144
Unique eGenes	1	0	1725

^a^Independent signals = independent AMD-associated genetic variants as defined by Fritsche et al.^2^

### Interaction of assigned variants in the lipid metabolism pathway

**Figure 3:**
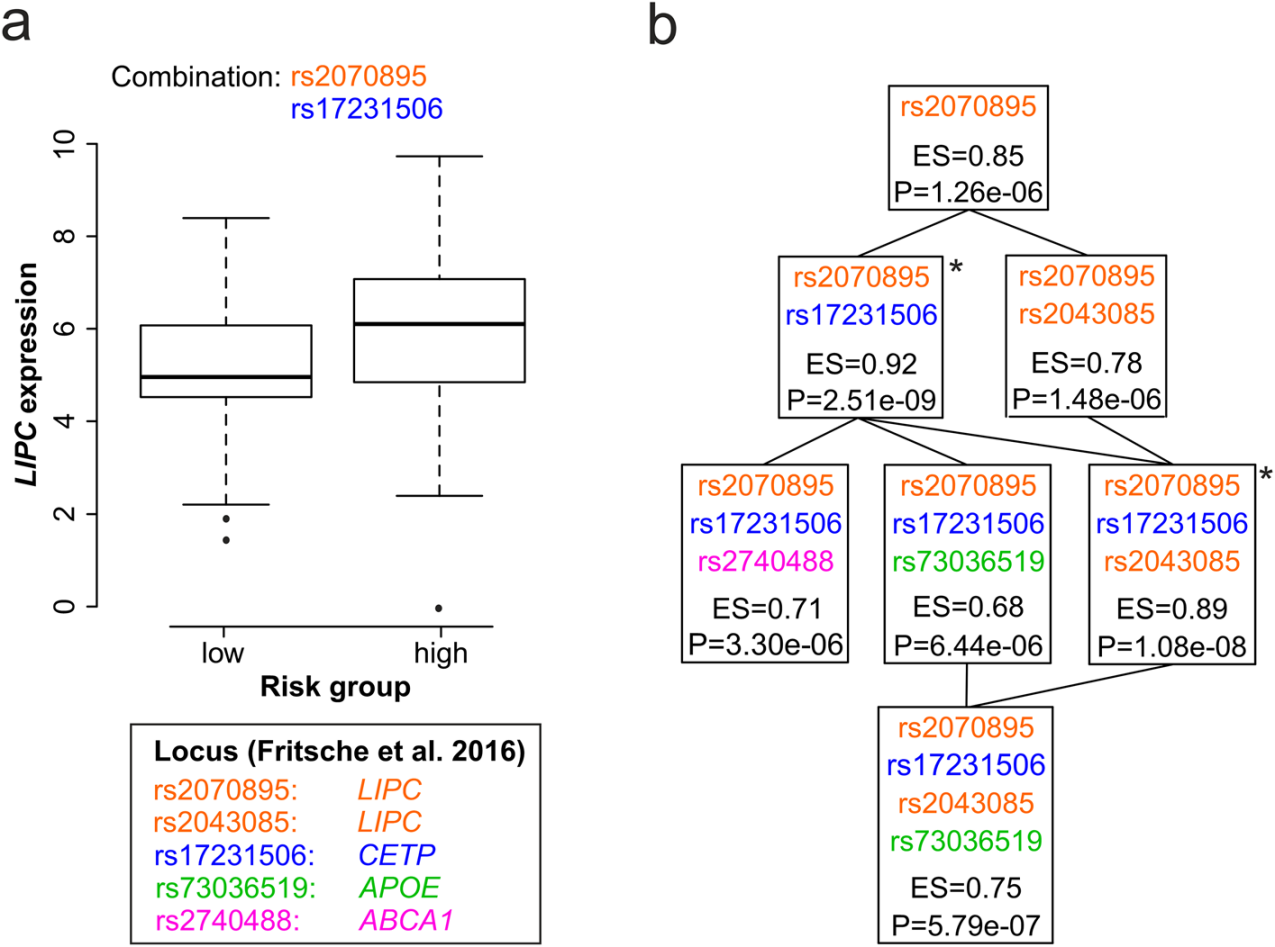
eCombinations altering expression of *LIPC*. (**a**) The boxplot compares *LIPC* expression between the AMD low and high-risk groups based on the genetic profile of the two genetic variants rs2070895 (orange, located in the *LIPC* locus) and rs17231506 (blue, located in the *CETP* locus). The y-axis corresponds to the relative level of gene expression. (**b**) The tree chart shows related variant combinations influencing the expression of *LIPC*. The chart starts with a single variant combination at the top and adds one variant per step downwards. Every box represents a set of variants used to determine risk groups. The P-value (P) and the effect size (ES) display the relationship between calculated risk groups and gene expression. Combinations highlighted with an asterisk remain significant after adjustment for multiple testing (Q-value < 0.05). Color code of variants refer to their locus affiliation.

We identified two eCombinations (rs2070895/rs17231506; rs2043085/rs2070895/rs17231506) in the lipid metabolism pathway significantly influencing gene expression in liver. Both eCombinations correlate with the expression of the *LIPC* gene (Supplementary Table S2). The eCombination with the highest significance include the variant rs2070895 (*LIPC* locus, independent signal 23.2 in ref.^2^) and rs17231506 (*CETP* locus) (Fig. 3a,b). Based on the two variants individuals with a high GRS showed a significantly increased expression of *LIPC*, in comparison to individuals with a low GRS (effect size (ES) = 0.92, standard error (SE) = 0.15, Q-value = 7.7 × 10^–3^). The second eCombination with a significant impact on *LIPC* expression includes the same variants as the first eCombination, and additionally rs2043085 (*LIPC* locus, independent signal 23.1 in ref.^2^). Again, individuals with a high GRS have a higher *LIPC* expression (ES = 0.89, SE = 0.15, Q-value = 1.65 × 10^–2^).

Next, we investigated additional eCombinations and their effect on gene expression differences between risk groups. We modified the combinations step-wise by adding or removing genetic variants. This identified less strong, and less significant effects (Fig. 3b). Of note, in our dataset the single variant rs2070895, which contributes to a known liver eQTL with *LIPC*^7^*,* revealed no significant effect after correcting for multiple testing.

### Interaction of assigned variants in the extracellular matrix pathway

Fifteen independent genetic variants were assigned to the extracellular matrix pathway (Fig. 1b), resulting in 32,767 unique combinations. We then analyzed the effect of each combination on the expression of 24,123 genes, which required over 790 million tests (Table 1). Applying the same threshold as described above, no significant correlation was observed (Q-value < 0.05) (Supplementary Table S3). This was confirmed by a quantile–quantile-plot, which showed no P-value differing from those expected by chance (Supplementary Figure S2b). Interestingly, the two eCombinations significant in the lipid metabolism pathway were included in this analysis, but failed to remain significant due to the higher burden of multiple testing.

### Interaction of assigned variants in the complement pathway

**Figure 4:**
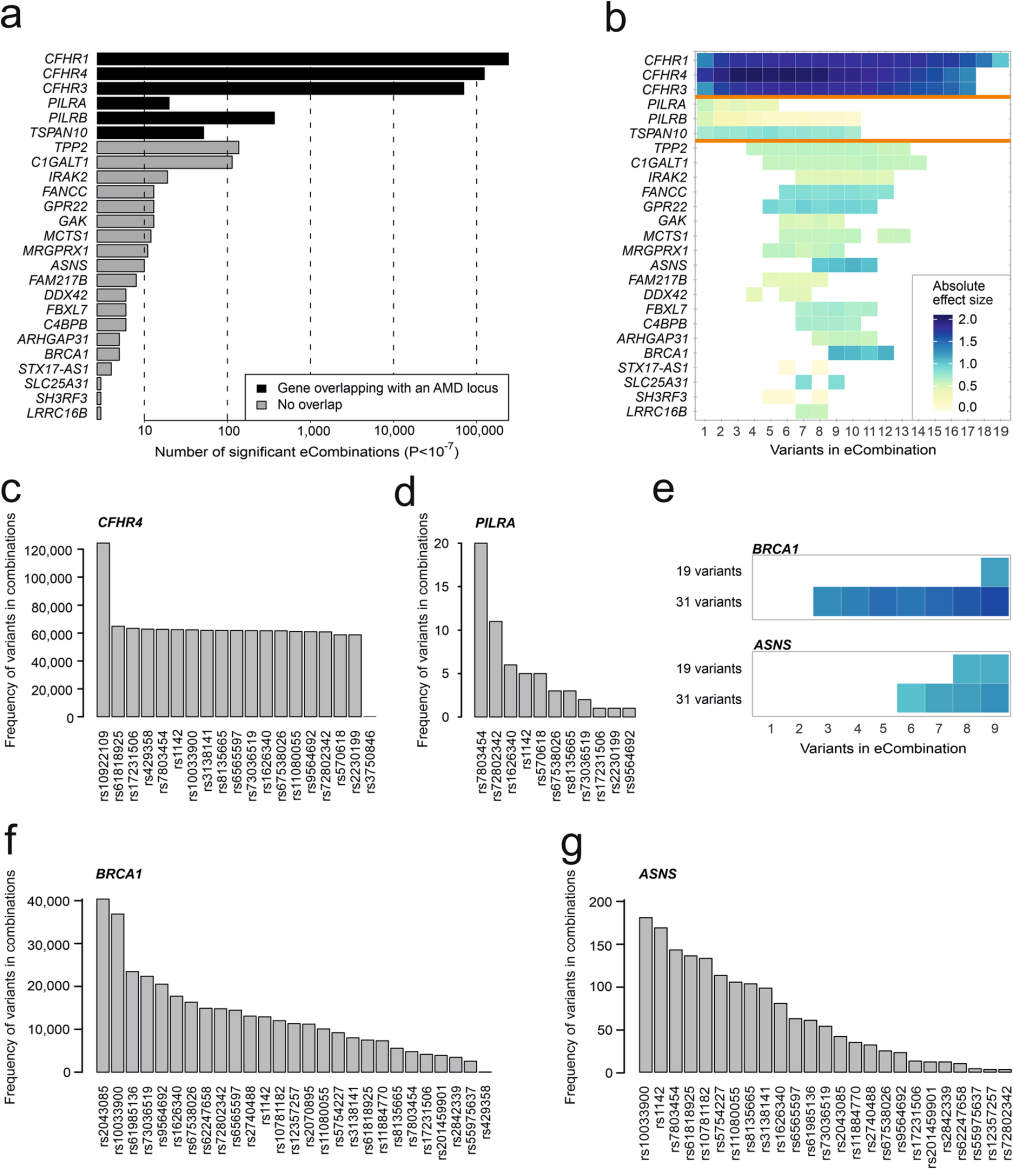
Interaction of genetic variants assigned to the immune pathway*.* (**a**) Number of eCombinations (P-value < 10^–7^) influencing the expression of the respective eGene given on the Y-axis. eCombinations were generated from genetic variants assigned to the immune pathway by Fritsche et al.^2^ and only eGenes which were detected at least three times are shown. Applying these thresholds, 438,219 significant results were obtained and related to expression changes of 25 eGenes. Black bars highlight eGenes that overlap with a known AMD locus^2^. (**b**) Color-coded effect sizes (ES) reflecting the correlation of eCombinations and gene expression. Colored fields show the absolute ES of an eCombination. White fields indicate a non-significant combination. The X-axis displays the number of genetic variants included in the respective eCombination. The orange line separates three subtypes of results, specifically one group including the *CFH*-related genes with one genetic variant being sufficient for eQTL detection and high absolute ES (highest absolute ES > 1.8), one group with a single genetic variant being sufficient for eQTL detection but lower ES (highest absolute ES < 1), and one group where an effect on gene expression can be observed by combining several genetic variants. (**c**,**d**) Contribution of different genetic variants to combinatory effects on gene expression of *CFHR4* (**c**, 124,372 eCombinations) and *PILRA* (**d**, 20 eCombinations). (**e**) Comparison of testing combinatory effects of all 31 AMD-associated variants and testing combinatory effects of 19 variants assigned to the category immune system process. The heatmap functionally refers to (**b**). (**f**,**g**) Contribution of different variants to combinatory effects on gene expression of *BRCA1* (**f**, 40,903 eCombinations) and *ASNS* (**g**, 192 eCombinations) with all 31 variants included in the analysis. Results refer to a P-value threshold < 10^–7^.

The 19 variants assigned to the complement pathway category result in 524,287 unique combinations which would require over 12 billion tests. A quantile–quantile-plot demonstrated that P-values smaller than 10^–5^ did not occur by chance (Supplementary Figure S2c). Altogether 641,016 significant results were observed, including 326,144 unique eCombinations and 1725 eGenes, whereby eCombinations could influence the expression of more than one eGene (Table 1, Supplementary Table S4). To focus on the most significant effects, the significance threshold was lowered to a P-value < 10^–7^ and only eGenes detected by at least three different eCombinations were analyzed (Supplementary Table S5). The filtering reduced the significant results to 438,219, including 243,302 unique eCombinations and 25 eGenes (Fig. 4a). Interestingly, over 99% of all significant results correspond to expression alterations of the complement factor H related genes 1, 3 and 4 (*CFHR1*: 242,652 eCombinations, *CFHR4*: 124,372 eCombinations, and *CFHR3*: 70,362 eCombinations). To further evaluate significant eCombinations, we initially focused on the number of variants included in the various combinations. This defined three categories of genes (Fig. 4b):

#### eGenes influenced by single variants and combinatory effects with almost any variant

This group refers to *CFH*-related genes CFHR1, CFHR3, and CFHR4, which also show the strongest differences in expression between low and high-risk individuals (highest absolute ES > 1.8) (Fig. 4b). To identify effect-driving variants, we examined how often each variant is represented among all eCombinations for the respective eGene (Supplementary Table S6). For example, *CFHR4* expression is significantly altered by 124,372 eCombinations, and each one of these combinations includes the AMD-associated variant rs10922109 (*CFH* locus) (Fig. 4c). In contrast, variant rs3750846 (locus *ARMS2*/*HTRA*) contributes to 103 of the 124,372 eCombinations while all other variants in this category contribute to approximately 50% of eCombinations, including variants rs570618 and rs61818925 located at the *CFH* locus (Supplementary Table S6).

#### eGenes influenced by single variants and combinations including a maximum of 10 variants

The second group of eGenes includes the genes *PILRA*, *PILRB*, and *TSPAN10*. Overall, the observed ESs are smaller than in the first group of eGenes (highest absolute ES < 1). A single variant (rs7803454, *PILRB/PILRA* locus) drives the strongest effect on expression of *PILRA* (ES = 0.66, SE = 0.09, Q-value = 9.21 × 10^–11^) and *PILRB* (ES = 0.59, SE = 0.04, Q-value = 7.97 × 10^–29^) (Supplementary Table S5). Also, this variant contributes to every eCombination identifying *PILRA* (Fig. 4d), although the ES decreases when additional variants join the eCombination (Fig. 4b). In contrast, an eCombination of 5 variants shows the strongest effect on *TSPAN10* expression (ES = − 0.93, SE = 0.14, Q-value = 2.77 × 10^–9^) (Fig. 4b).

#### eGenes exclusively influenced by combinatorial effects

Nineteen eGenes within the third group reveal changes in gene expression due to eCombinations of at least 4 and a maximum of 14 variants (Fig. 4b). *BRCA1* and *ASNS* show the highest ESs in this group with downregulation of *BRCA1* (ES = − 1.18, SE = 0.21, Q-value = 1.55 × 10^–7^; 12 variants), and upregulation of *ASNS* (ES = 1.17, SE = 0.20, Q-value = 9.36 × 10^–8^; 10 variants) (Supplementary Table S5).

### Validation of regulatory effects of variants assigned to the complement pathway

To validate the methodological approach, we established a two-step protocol based on data of the genotype-tissue expression (GTEx) project. In a first step, we tested whether our findings are liver-specific. Consequently, we selected the five eCombinations with the strongest absolute ES for the 25 eGenes identified (Fig. 4a) and analyzed their regulatory effect on the respective gene in three tissues with comparable sample sizes to the liver data, namely skeletal muscle (584 samples), whole blood (556 samples) and subcutaneous adipose tissue (477 samples) (Supplementary Table S7). Out of the 25 genes, four (*CFRH4*, *SLC25A31*, *GPR22* and *MRGPRX1*) were not expressed in the selected tissues. Of the remaining 21 genes, the regulatory effects on genes known for their local eQTL (*CFHR1*, *CFHR3*, *PILRA*, *PILRB* and *TSPAN10*) were regularly detectable with the exception of *PILRA* in whole blood for any of the tested eCombinations including the single eVariant rs7803454 (representing a local eQTL). Regulatory effects on the remaining genes, which are not known for their local eQTL and mostly exhibited smaller ESs in the initial analysis in liver tissue, were observed only sporadically in the three additional tissues examined, and not always with the same effect direction. This indicates that the observed regulatory interaction effects of AMD-associated variants on gene expression represent mainly tissue-specific effects.

The second validation step applied an undirected approach. Essentially, we repeated the interaction analysis to identify eGenes influenced by interaction of complement pathway assigned variants in the GTEx tissue skeletal muscle. Applying a threshold of P < 10^–5^, we observed 160,466 significant results, including 102,293 unique eCombinations and 1425 unique eGenes (Supplementary Table S8). A direct comparison with the results obtained at the same threshold in liver tissue (P-value < 10^–5^, Supplementary Table S4) revealed 80 eGenes identified in both tissues (out of 1725 unique eGenes in liver and 1425 unique eGenes in muscle). Moreover, screening for the eGenes with the most robust effects in liver (25 eGenes, P-value < 10^–7^ and detected by at least three eCombinations, Supplementary Table S5), three eGenes were also detected in muscle (Supplementary Table S8), namely *PILRA*, *PILRB* and *IRAK2*. While *PILRA* and *PILRB* both represent eGenes with known strong local eQTL effects, *IRAK2* was solely detected in liver when a risk profile including 7 to 12 AMD-associated variants was examined (Supplementary Table S5, Fig. 4b). In muscle, a regulatory effect on *IRAK2* was detected by 4 eCombinations, including 4 to 6 variants (Supplementary Table S8).

Applying the same critical thresholds for muscle tissue as was done for liver to address the strongest effects (P-value < 10^–7^ and only eGenes detected by at least three eCombinations), 41,206 significant results were obtained, including 17,836 unique eCombinations and 17 unique eGenes (Supplementary Table S9). Comparing these results with liver data for the same thresholds (Supplementary Table S5), identified two eGenes detected in both tissues, namely *PILRA* and *PILRB*.

Together, the methodological validation demonstrates that the combination of GRS and eQTL analyses represents a valid approach to investigate regulatory effects of a defined genetic risk profile on gene expression in independent datasets and multiple tissues.

### Resolution of regulatory effects on *BRCA1* and *ASNS* expression in liver

The combination of GRS and eQTL analyses has the power to identify novel potentially disease-relevant eGenes based on epistatic effects of available eCombination data. In liver tissue strongest effects for novel AMD eGenes were observed for *BRCA1* and *ASNS*. These genes become evident when testing variant combinations for a joint effect on gene expression within the complement pathway. There is currently no known involvement of the *BRCA1* or *ASNS* gene in this pathway, or more specifically in AMD pathology. To gain a deeper insight by refining the risk profile required for a change of expression, we assessed combinations of all 31 AMD-associated genetic variants included in our dataset (Fig. 4e) and restricted testing for combinations consisting of a maximum of 9 variants due to the large computational and multiple testing burden. A P-value threshold of < 1 × 10^–7^ resulted in 40,903 eCombinations with impact on *BRCA1* expression (Supplementary Table S10), and 192 eCombinations with impact on *ASNS* expression (Supplementary Table S11). Interestingly, the analysis including the 31 variants demonstrates even stronger ESs when compared with the results exclusively obtained with the complement-related variants (Supplementary Table S5). For example, *BRCA1* expression reveals an ES of − 1.61 (SE = 0.22, Q-value = 5.04 × 10^–6^) in contrast to the initially strongest ES of − 1.18 (SE = 0.21, Q-value = 1.55 × 10^–7^). *ASNS* also shows a small increase of ESs from the initially strongest ES of 1.17 (SE = 0.20, Q-value = 9.36 × 10^–8^) to an ES of 1.25 (SE = 0.20, Q-value = 8.99 × 10^–6^) (Fig. 4e).

Regarding *BRCA1* expression, our analysis identifies two effect-driving variants with rs2043085 (*LIPC* locus) in 98.7% (40,366) and rs10033900 (*CFI* locus) in 90.2% (36,873) of the 40,903 eCombinations (Fig. 4f). 25 variants appear less often (0.10–57.31%) and 4 variants do not contribute to any significant eCombination, namely rs10922109 (*CFH* locus), rs570618 (*CFH* locus), rs3750846 (*ARMS2/HTRA1* locus) and rs2230199 (*C3* locus). Interestingly, the odds ratios (ORs) in the AMD GWAS of contributing variants vary between 1.09 and 1.49 while the ORs of non-contributing variants vary between 1.47 and 2.93^2^.

*ASNS* expression is mostly driven by eCombinations containing rs10033900 (*CFI* locus) (182 eCombinations, 94.8%), followed by rs1142 (*KMT2E*/*SRPK2* locus) (170 eCombinations, 88.54%) (Fig. 4g). The frequency of the other 23 variants in the eCombinations vary between 75% (144 eCombinations) and 1.56% (3 eCombinations). Six variants exert no influence on *ASNS* expression, including rs2070895 (*LIPC* locus) and rs429358 (*APOE* locus) as well as the same 4 variants which also do not contribute to *BRCA1* expression.

## Discussion

Within the past 15 years, GWAS have deepened our knowledge on the genetic factors conferring risk to develop complex phenotypes. Still, enormous challenges remain to better understand the complexities and interactions of the genetic contributions to disease. Specifically, functional consequences arising from multiple genetic variants, individually often contributing only small effects, need to be addressed. The latter issue is key insofar as it reflects a situation where a person almost always harbors various combinations of multiple risk variants. With this in mind, we aimed for an approach which tests epistatic effects of independent GWAS variants associated with AMD. We used variant combinations to generate genetic risk models in a dataset of 588 individuals with unknown AMD status. The genetic information was then correlated with gene expression in a model tissue, namely liver. This approach revealed 26 genes with a significantly altered expression in high-risk versus low-risk variant combinations. Of these, seven eGenes, namely *LIPC*, *CFHR1*, *CFHR4*, *CFHR3*, *PILRA*, *PILRB*, and *TSPAN10* were previously reported to be regulated by single AMD-associated variants^7^ while 19 eGenes represent exciting new candidates becoming evident only due to additive effects of AMD-associated genetic variant combinations.

Choosing three biologically relevant pathways in AMD^2,6,14,20^, we interrogated previous GWAS data to determine 21 functionally associated loci corresponding to 31 independent genetic variants. Testing all possible combinations would have generated a multiple testing burden of over 52 trillion tests, likely obscuring true positive signals^22^. Even reducing the number of genetic variants by pathway allocation only remotely reduces the impact of multiple testing. For example, the analysis of seven variants within the lipid metabolism pathway-related loci required around 3 million tests which, after correction for multiple testing, resulted in two eCombinations correlated with *LIPC* expression. Already the combination of 15 variants located in extracellular matrix pathway-related loci resulted in 790 million tests and revealed no significant result, even though all combinations of the lipid metabolism pathway approach were included in this analysis. This demonstrates the need to initially define a precise hypothesis to keep the number of tests within a reasonable magnitude consequently avoiding false-negative results due to excessive test corrections.

The correlation of *LIPC* expression and AMD pathology was established before^7,23^. One study showed an increased *LIPC* expression in AMD by applying a machine learning approach known as PrediXcan, which predicts gene expression based on genetic variation^23^. This approach, however, does not allow a resolution of the association down to a distinct variant. In a second study applying eQTL analysis in liver tissue, *LIPC* expression was directly correlated with local risk variant rs2070895^7^. Interestingly, the current analysis revealed a significant correlation between an increased *LIPC* expression and a risk genotype containing rs2070895 (*LIPC* locus, chromosome 15q21.3) and rs17231506 (*CETP* locus, chromosome 16q13). The combination of these two variants displayed an even higher ES than rs2070895 alone. The mechanism of the combined effect of these two variants on *LIPC* expression needs to be clarified, specifically since *LIPC* and *CETP* are attributed to distinct processes within the lipid metabolism pathway^7,24–26^.

Gene expression regulation by genetic variants at immune-related loci initially revealed a remarkably high number of over 1700 eGenes. We therefore focused on the most significant results with P-value < 1 × 10^–7^ and filtered for eGenes which are correlated with at least three eCombinations leaving 25 eGenes of interest. Remarkably, three of these 25 eGenes were also detected by applying our two-step approach in an independent dataset of skeletal muscle tissue from the GTEx project. This suggests that combining GRS and eQTL analyses yield valid and robust results. Six of the eGenes identified in liver have been linked to AMD before and are well-known eQTL genes, namely *CFHR1*, *CFHR3* and *CFHR4*, as well as *PILRA*, *PILRB*, and *TSPAN10*^6,7,23,27^. The major effect driving variant for the three *CFH*-related genes is rs10922109 which is located within the *CFH* locus. The addition of further variants to the genetic risk model reveals only little impact on ESs. In fact, including rs3750846, a variant localized within the *ARMS2/HTRA1* locus, eliminates the effect on the expression of *CFH*-related genes. This could suggest that *CFH*-related genes and relevant genes in the *ARMS2/HTRA1* interval are involved in independent mechanisms leading to AMD pathology.

Of the 26 eGenes with differential expression in high-risk versus low-risk variant combinations, 19 eGenes have not been associated with AMD pathogenesis before. We speculate that the novel findings are the result of a summation of small unidirectional effects of single genetic variants. Of note, the corresponding variants are often located in distant (trans) loci, such as eCombination rs2043085 (chromosome 15) and rs10033900 (chromosome 4), which in combination with further variants influence the expression of *BRCA1* (chromosome 17). Some of such distant variant combinations have ESs on gene expression that are even stronger than previously reported cis-eQTL variants^7^. Of note, a validation of our approach in different tissues available in the GTEx dataset highlight that most of the 19 eGenes associated with AMD for the first time, likely represent liver-specific findings.

Following our eCombination risk approach, the unexpected findings for *BRCA1* and *ASNS* expression were substantiated by generating combinations from the full set of 31 independent AMD-associated variants included in our dataset. Considering the burden of multiple testing, we explored a maximum of nine variants per combination. This resulted in even stronger effects than in our initial findings which were limited to results from loci pre-assigned to the complement pathway. Loss of function mutations in *BRCA1* are foremost known to be correlated with an increased risk to develop breast and ovarian cancer^28^. Functionally, BRCA1 is involved in repairing double strand breaks but has also been reported to regulate telomere length and stability^29^. Shortened telomere length is related to cell senescence, and double strand breaks are considered to be highly relevant in aging^30,31^. As age is a major risk factor for the development of AMD, this is a potential confounder which was adjusted for in the current study. Our data now point to downregulation of *BRCA1* expression as part of the AMD pathomechanism which is further supported by a previous study that implicated DNA damage and a reduced DNA repair potential in AMD etiology^32^. Another remarkable finding in our analysis relates to the eGene *ASNS*, whose protein product catalyzes the conversion of aspartate to asparagine in the presence of glutamine^33^. A recent study examined the role of asparagine and glutamine in angiogenesis, and demonstrated that silencing of *ASNS* inhibited endothelial cell sprouting^34^. Our data now demonstrate that an increased AMD risk is significantly correlated with an upregulation of *ASNS* expression. This could make ASNS a pro-angiogenic factor possibly involved in neovascular complications of late-stage AMD.

It is important to note that the present study is not suited to identify novel genetic variants associated with AMD as conventional GWAS usually do. In fact, our approach facilitates the detection of effects on so far undetected gene expression that only becomes apparent if an individual carries a distinct combination of known AMD-associated risk variants. This represents a unique approach to find disease-associated genes that are potentially hidden in existing GWAS data. It allows the identification of individuals at risk who might especially benefit from a certain prevention strategy or therapy. Although most of our findings show small to moderate ES, the altered regulation of gene expression likely exerts a lifetime effect and it is conceivable that the effects on gene expression even increase in combination with other factors, like ageing or smoking.

Taken together, our study presents a novel approach to investigate joint effects of genetic variants on gene expression by combining GRS and eQTL mapping. We replicate a number of previous eQTL findings in AMD GWAS data, and report 19 novel genes correlating with the genetic risk to develop AMD. All genes were identified by jointly analyzing several seemingly independent AMD-associated signals, which is perfectly in-line with the idea that the signals underlying GWAS associations contribute to shared biological mechanisms.

## Methods

### AMD risk variants and dataset

52 independent genetic variants associated with AMD and their respective ORs were extracted from the most recent GWAS dataset^2^. Genotype and gene expression data of liver tissue from 588 individuals of European ancestry were provided by Strunz and colleagues^7^. The latter study combined data from four different studies^35–38^ and contains expression information on 24,123 genes. From genotype data, variants with (1) unavailable information, (2) a minor allele frequency less than 5% and (3) multi-allelic variants, were excluded. Thus, genotypes of 31 of the 52 AMD-associated variants were available for our study (Supplementary Table S1).

### Category definition

The independent AMD variants were assigned to three superordinate biological processes according to biological pathways relevant in AMD pathology. To this end, 368 genes located in AMD loci as defined by Fritsche and colleagues (index variant and proxies (r^2^ > 0.5 and ± 100 kb) were included^2^. Functional profiling^21^ of these genes revealed 48 significantly enriched biological processes (g:SCS algorithm corrected for multiple testing, user threshold: 0.05). The superordinate biological processes were detected by connecting all significant processes in ancestor charts (https://www.ebi.ac.uk/QuickGO/, accessed November 2019). Two processes, “Cholesterol transport” and “Regulation of plasma lipoprotein”, were located in independent ancestor trees and were merged to one category (“lipid metabolism pathways”) due to their high similarities. An AMD locus was assigned to a category when at least one gene overlapping with the locus was assigned to the respective biological process.

### Variant combination and GRS calculation

To generate the variant combinations, we used the R^39^ function *combn*^39^. Every combination consists of a sequence of variants that is unique in its composition. The number of variants in a combination varies between 1 and the total number of variants used for calculation. For every combination of variants a unique GRS was calculated as reported elsewhere^40^. In short, the GRS is defined as the sum of risk alleles of one individual weighted by the respective ES of each variant, which was obtained from the latest AMD GWAS^2^. As a consequence, the OR was transformed by the natural logarithm. Instead of centering the GRS, we normalized it by dividing it through the average ES of all included variants as reported elsewhere^13^.

### Determination of risk group sizes

To determine the risk group sizes, all seven variants assigned to the superordinate biological process “Cholesterol transport/Regulation of plasma lipoprotein” were used to generate a GRS. According to their GRS, individuals were classified into risk groups. To determine the size of the risk groups ensuring sufficient power for subsequent tests, we performed eQTL analysis in low and high-risk groups with different group sizes (5%, 10%, 15%, 20%, 25%, 30% and 35%) of the entire dataset. eQTL analysis were performed with Matrix eQTL^41^. The group size was regarded to be sufficient in cases in which a replication could be shown for four previously detected eGenes, specifically *LIPC*, *CETP*, *ALDH1A2* and *ADAM10*, in the same tissue^7^ with a FDR^42^ smaller than 5%. A proportion of 30% (176 samples) of the total cohort size (588 samples) was determined to be the minimal group size (Supplementary Figure S1).

### Regression analysis, adjustment for multiple testing and trans-eQTL analysis

A multivariate linear regression was performed using *Matrix eQTL*^41^ to examine the relationship between AMD risk group and gene expression. The covariates age, gender, the original study representing the data source, and the first five genotype based principle components were included as provided by Strunz and colleagues^7^. The variants included in each initial category were analyzed in independent test series. We adjusted for multiple testing in every test series separately by controlling the FDR to be smaller than 5%. The FDR was calculated using the R function *p.adjust* (method “fdr”)^39^.

### Validation of approach in GTEx

To validate our combined approach of GRS and eQTL, we used the genotype and gene expression data of the GTEx project (version 8). Whole genome sequencing data were retrieved from dbGaP (accession ID: phs000424.v8.p2) in VCF format^43^. Detailed information about the genotype processing and quality control (QC) protocols are provided elsewhere^44^. To determine ethnicity of samples, a principal component analysis (PCA) was carried out in R (version 3.3.1)^45^ using the *snpgdsPCA*^46^ function based on 100,000 random genetic variants of each sample and the corresponding genotype information of the 1000 Genomes Project reference panel (Phase 3, release 20130502)^47^. The first two principal components were plotted to determine the ethnicity. Only samples clustering next to the European reference individuals were included to consider the known variation of haplotype structures between populations. Gene expression and covariate data of three tissues were downloaded from the GTEx Portal^48^ and filtered for European individuals based on the genotype PCA. Altogether, genotype, gene expression, and covariate data from 584 muscle skeletal, 556 whole blood, and 477 adipose subcutaneous samples were included in the validation step. The AMD-associated variant rs61818925 (AMD signal 1.6)^2^ was not covered in the GTEx dataset and was replaced by the proxy rs61818924, which shows a R^2^ to rs61818925 of 0.8 in Europeans.

## Supplementary Information

Supplementary Information 1.

Supplementary Information 2.

## Data Availability

Genotype and gene expression data of the liver eQTL dataset were provided by Strunz and colleagues and are available in public databases as reported^7^. GWAS information for genetic variants, locus names and ORs were taken from Fritsche et al.^2^. Genotype, gene expression, and covariate data of the GTEx project are available in dbGaP (accession ID: phs000424.v8.p2) or the GTEx portal (http://www.gtexportal.org).
