# Supplementary material for: Epistatic interactions of genetic loci associated with age-related macular degeneration
Source: Sci Rep. 2021 Jun 23;11:13114. doi: 10.1038/s41598-021-92351-4 (PMC8222216; doi:10.1038/s41598-021-92351-4)
Supplement: Supplementary file 1 — Supplementary Information 1. [file 41598_2021_92351_MOESM1_ESM.pdf]

Supplementary information

EPISTATIC INTERACTIONS OF GENETIC LOCI ASSOCIATED WITH AGE-RELATED MACULAR DEGENERATION

Christina Kiel, Christoph A. Nebauer, Tobias Strunz, Simon Stelzl, Bernhard H. F. Weber

This supplementary information file contains

- Supplementary Tables S1 to S11
- Supplementary Figures S1 to S2 including figure legends

Supplementary Table S1. Categorization of AMD-associated variants.

Supplementary Table S2. eGenes influenced by interaction of lipid pathway assigned variants.

Supplementary Table S3. eGenes influenced by interaction of extracellular matrix pathway assigned variants.

Supplementary Table S4. eGenes influenced by interaction of complement pathway assigned variants.

Supplementary Table S5. Most robust eGenes influenced by interaction of complement pathway assigned variants.

Supplementary Table S6. Contribution of variants assigned to the complement pathway to eCombinations.

Supplementary Table S7. Validation of eGenes influenced by interaction of complement pathway assigned variants in selected genotype-tissue expression (GTEx) project tissues.

Supplementary Table S8. eGenes influenced by interaction of complement pathway assigned variants in the GTEx project tissue muscle skeletal.

Supplementary Table S9. Most robust eGenes influenced by interaction of complement pathway assigned variants in the GTEx project tissue muscle skeletal.

Supplementary Table S10. eCombinations influencing *BRCA1* expression.

Supplementary Table S11. eCombinations influencing *ASNS* expression.

Supplementary Tables S1 to S11 have been combined in "Supplementary_Tables S1 to S11.xlsx" (76.1 Mb)

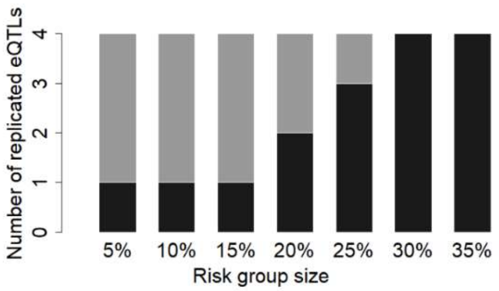

Supplementary Figure S1. Determination of risk group size. Risk group size was determined to be 30 % (176 samples) of the total cohort size (588 samples). In order to determine the risk group size that ensures sufficient power for later tests we performed eQTL analysis in low and high-risk groups with different group sizes. The group size was defined to be sufficient if a replication of four previously detected eQTL[2] with a FDR[3] smaller than 5 % was possible. For risk group determination, a combination of all seven variants assigned to the lipid metabolism pathway category was used. The x-axis shows different risk group sizes given in percentage. The black bars indicate the number of replicated eQTL for the respective risk group size.

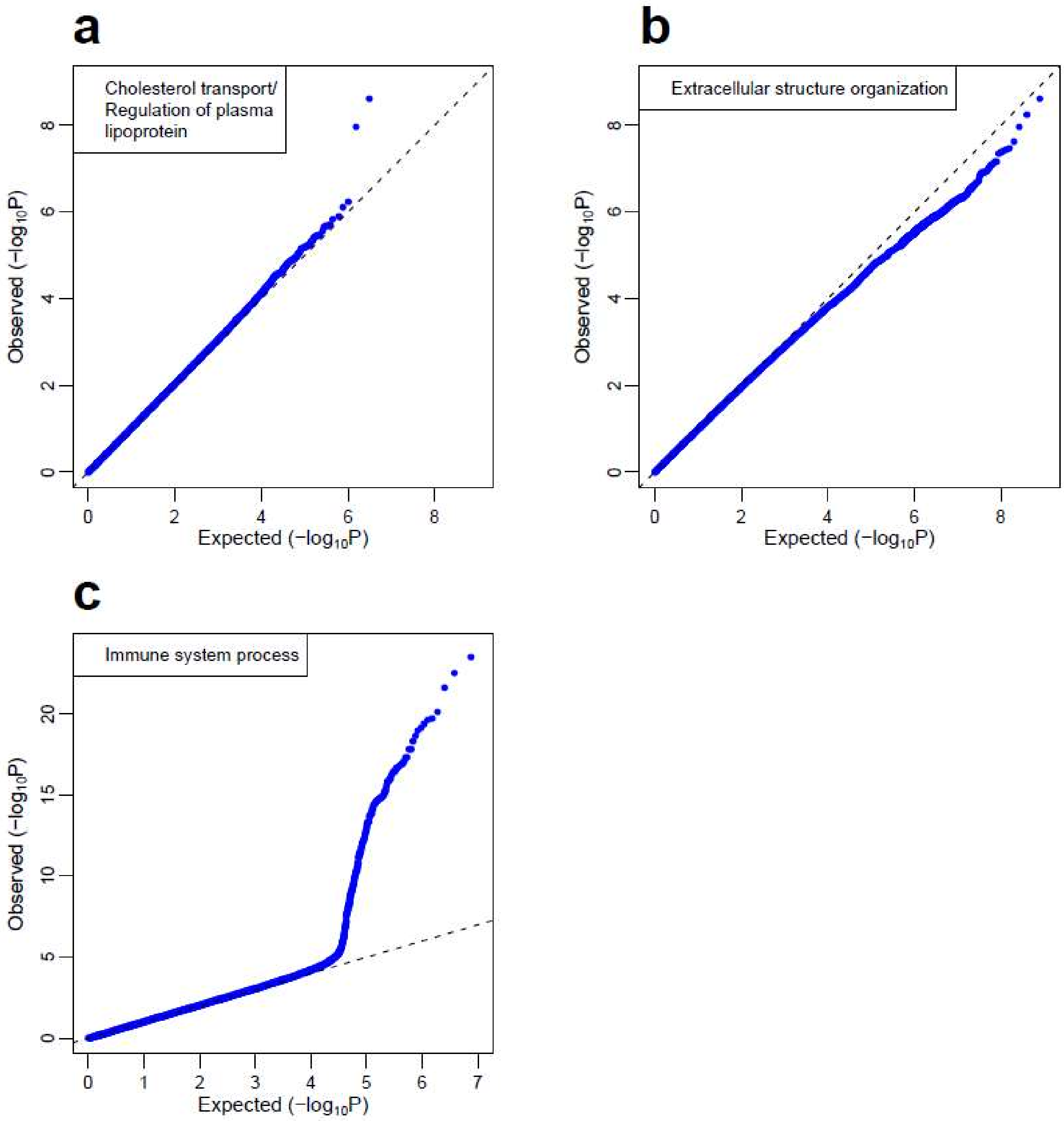

Supplementary Figure S2. Quantile-quantile-plots for correlation analysis of genetic risk profiles and gene expression levels. Shown are the expected (x-axis) and observed (y-axis) P-values of each tested category. Quantile-quantile plots show which P-values differ from those expected under the null hypothesis (no significant linear regression). **(a)** Cholesterol transport/regulation of plasma lipoprotein category includes 3 million tests. **(b)** Extracellular structure organization category includes 790 million tests, 8.5 million tests are represented. **(c)** Immune system processes category includes 12.6 billion tests, 7.6 million tests are represented.
